# Automated Measurement of Net Water Uptake From Baseline and Follow-Up CTs in Patients With Large Vessel Occlusion Stroke

**DOI:** 10.3389/fneur.2022.898728

**Published:** 2022-06-27

**Authors:** Atul Kumar, Yasheng Chen, Aaron Corbin, Ali Hamzehloo, Amin Abedini, Zeynep Vardar, Grace Carey, Kunal Bhatia, Laura Heitsch, Jamal J. Derakhshan, Jin-Moo Lee, Rajat Dhar

**Affiliations:** ^1^Department of Neurology, Washington University in St. Louis School of Medicine, Saint Louis, MO, United States; ^2^Saint Louis University School of Medicine, Saint Louis, MO, United States; ^3^Department of Radiology, Washington University in St. Louis School of Medicine, Saint Louis, MO, United States; ^4^Department of Radiology, University of Massachusetts Medical School, Worcester, MA, United States; ^5^Department of Emergency Medicine, Washington University in St. Louis School of Medicine, Saint Louis, MO, United States

**Keywords:** stroke, cerebral edema area, computed tomography, machine learning, image segmentation

## Abstract

Quantifying the extent and evolution of cerebral edema developing after stroke is an important but challenging goal. Lesional net water uptake (NWU) is a promising CT-based biomarker of edema, but its measurement requires manually delineating infarcted tissue and mirrored regions in the contralateral hemisphere. We implement an imaging pipeline capable of automatically segmenting the infarct region and calculating NWU from both baseline and follow-up CTs of large-vessel occlusion (LVO) patients. Infarct core is extracted from CT perfusion images using a deconvolution algorithm while infarcts on follow-up CTs were segmented from non-contrast CT (NCCT) using a deep-learning algorithm. These infarct masks were flipped along the brain midline to generate mirrored regions in the contralateral hemisphere of NCCT; NWU was calculated as one minus the ratio of densities between regions, removing voxels segmented as CSF and with HU outside thresholds of 20–80 (normal hemisphere and baseline CT) and 0–40 (infarct region on follow-up). Automated results were compared with those obtained using manually-drawn infarcts and an ASPECTS region-of-interest based method that samples densities within the infarct and normal hemisphere, using intraclass correlation coefficient (ρ). This was tested on serial CTs from 55 patients with anterior circulation LVO (including 66 follow-up CTs). Baseline NWU using automated core was 4.3% (IQR 2.6–7.3) and correlated with manual measurement (ρ = 0.80, *p* < 0.0001) and ASPECTS (*r* = −0.60, *p* = 0.0001). Automatically segmented infarct volumes (median 110-ml) correlated to manually-drawn volumes (ρ = 0.96, *p* < 0.0001) with median Dice similarity coefficient of 0.83 (IQR 0.72–0.90). Automated NWU was 24.6% (IQR 20–27) and highly correlated to NWU from manually-drawn infarcts (ρ = 0.98) and the sampling-based method (ρ = 0.68, both *p* < 0.0001). We conclude that this automated imaging pipeline is able to accurately quantify region of infarction and NWU from serial CTs and could be leveraged to study the evolution and impact of edema in large cohorts of stroke patients.

## Introduction

A major consequence of brain ischemia is the development of cerebral edema. This water accumulation within and around the injured tissue leads to brain swelling, raising compartmental pressure and eventually leading to midline shift and herniation. The development of malignant cerebral edema represents the greatest source of mortality in the acute period after ischemic stroke, especially for strokes due to large vessel occlusion (LVO) (1). As key mediators remain incompletely understood, few interventions currently exist to mitigate cerebral edema (2). One of the major limitations in studying edema is the need for an accurate means of quantifying its formation in the early stages after stroke (3, 4). Midline shift is a crude measure that does not adequately capture edema as it develops over the first 24–48 h after stroke, but only captures its delayed and decompensated phenotype. Furthermore, labeling edema only when it leads to deterioration (i.e., malignant edema) obscures a continuum of injury severity that is seen across almost all LVO stroke patients (5).

One of the hallmarks of evolving brain edema is tissue hypoattenuation (6). This can be captured by the progressively decreasing density (measured in Hounsfield Units, HU) of infarcted tissue on non-contrast computed tomography (NCCT) imaging. NCCT is readily available and routinely performed in almost all stroke patients, both acutely on presentation and frequently at follow-up. It affords an accessible means of serially assessing edema as it develops in the days after stroke. However, measurement of the total lesional hypodensity volume encompasses both infarcted tissue and associated edema, with relative proportions varying across patients (5, 7). A recent imaging method has been proposed to disentangle the contribution of edema to subacute lesion volume and quantify the progression edema after stroke (8). *Net water uptake* (NWU) evaluates the relative density of the ischemic tissue compared to a contralateral homologous region; increasing NWU on admission NCCT has been associated with longer time from stroke onset to imaging and poor collateral status (9, 10). NWU has also exhibited promise in quantifying edema progression, rising more in those with malignant outcomes and in those without successful recanalization (11, 12). Therefore, it has emerged as one of the most promising biomarkers of edema after stroke, with a wide array of potential applications across LVO cohorts (13).

However, implementation of NWU measurement from serial CTs in large stroke cohorts faces several challenges. The principal challenge is that its assessment is dependent on identification and delineation of the area of early infarction on acute and subacute CTs. As this region is not usually clearly visible on baseline NCCT within a few hours of stroke onset, most studies measuring early NWU have relied on CT perfusion (CTP) images to visually guide manual delineation of core infarct. In some studies where CTP was not available, NWU was estimated by measuring density within regions-of-interest (ROIs) placed within ASPECTS regions exhibiting early hypoattenuation and matched regions in the contralateral hemisphere (14). Measurement of NWU on follow-up NCCT requires manually outlining the visible region of infarction and flipping this manual ROI to create a homologous normal region for density assessment. This approach is time-consuming, subject to variability, and makes studying edema in large cohorts with NWU, although attractive in theory, challenging to perform in practice. Our objective was to develop an accurate means of automatically extracting infarct regions and measuring NWU from both baseline and follow-up CTs of LVO stroke patients. This imaging algorithm could then be leveraged to accelerate research into edema using larger cohorts of stroke patients (15).

## Methods

### Study Participants

We evaluated patients undergoing stroke assessment at a single institution between May 2018 and November 2019 who underwent multimodal CT (NCCT with CT angiography and CTP) on presentation. We selected those who had evidence of LVO in the anterior circulation, affecting either the internal carotid artery (ICA) or proximal segment of the middle cerebral artery (MCA). We limited our analysis to those with measurable infarct core on baseline CTP (as assessed using the RAPID software package, iSchemaView, Redwood City, California). Approval from the institutional review board was obtained for a waiver of participant consent given the retrospective, de-identified nature of this research.

### Imaging Analysis

We collected all NCCT and CTP images performed at baseline as well as all follow-up NCCTs performed within 1 week of stroke onset. The CTP images were processed using an in-house algorithm that automatically extracts high-resolution image maps of perfusion parameters, including cerebral blood flow (CBF) and time to maximum (Tmax), as described in the Supplementary Methods and outlined in Supplementary Figure 1. Core infarct regions were defined by tissue with CBF below 30% of normal, where normal brain was defined as tissue with Tmax below 4 s (16). To assess consistency of core extraction, the volume of this core region was compared to the volume extracted by the RAPID software.

All NCCTs at baseline and follow-up were processed using an automated analysis workflow that is fully described in the Supplementary Methods. Key steps include: (i) registration of an atlas template with midline delineated on all slices to the target NCCT (17); (ii) segmentation of cerebrospinal fluid (CSF) regions using our well-established deep learning model (18, 19); and (iii) segmentation of visible acute infarct regions using a novel deep learning-based algorithm. This algorithm employed a deep learning model based on the U-Net architecture and previously trained for CSF segmentation (full details of this fully convolutional neural network provided in Supplementary Methods) (20). This network was further trained on 335 manually outlined infarct regions defined on NCCTs from a prior three institution stroke cohort as ground-truth labels. This training set was divided into ~90% (304 infarcts) for training and the remainder (21) for internal validation. The infarct region proposed by this algorithm was further refined by identifying the largest connected region in three dimensions as the likely primary infarct territory and excluding smaller disconnected voxels. The algorithm was then applied to all NCCTs in this cohort, including a test cohort of 28 subjects where the infarct region was manually segmented on all slices. The Dice Similarity Coefficient (DSC), a stringent metric of spatial overlap of voxels between the automated infarct region and the manually outlined ground-truth was calculated as twice the union of the two regions divided by the sum of their volumes.

This infarct region was utilized as the infarct mask for evaluating NWU on follow-up NCCTs, as well as for any baseline NCCTs on which infarct was already clearly visible. For the remainder of baseline CTs, the core regions from CTP analysis were utilized in place of infarct masks. These CTP-defined core masks were registered to the NCCT using FLIRT from FSL (FMRIB, Oxford, UK) (22). The brain was divided into hemispheres, using the registered midline, as previously applied to measure the hemispheric CSF ratio (23). The infarct region was then flipped along the brain midline to generate a homologous region in the contralateral hemisphere (as shown in Supplementary Figure 2).

For baseline CTs, we also instituted an automated patch-based approach for estimating NWU (that does not require CTP), similar to one recently proposed, to allow comparison with our full-infarct method (24). For this approach, square standard patches were placed on four separate axial slices within each MCA territory of a CT atlas and then this atlas was registered to each patient's baseline NCCT, allowing estimation of NWU from the density within the ischemic MCA territory compared with the contralateral side (Supplementary Figure 3).

All regions of CSF were removed from both infarct and mirror regions and thresholding was applied to remove voxels with HU outside an established range, initially set at 20–80, per prior protocols for NWU measurement (12). However, on review of the distribution of densities within regions of infarction on follow-up CTs [both from the literature (25) and our own data], we determined that expanding the lower limit to 0 HU would better capture lower density voxels that would otherwise be excluded if using a threshold of 20 (leading to an underestimation of NWU). We also chose to restrict the upper limit of infarct regions to 40 HU rather than 80; we found that most voxels within bland infarcts had densities below 40 HU while regions of hemorrhagic transformation often extended above 40 HU; this is supported studies of HU within intracerebral hematomas and should also exclude regions of contrast staining within evolving infarcts, specifically defined by HU > 40 (26, 27). Therefore, removing voxels with density above 40 HU from within the infarct would avoid contamination of NWU calculations by regions of HT and contrast staining, both issues that has confounded prior studies of NWU (28). The standard 20–80 threshold was used for the normal brain region and for the CTP core mask at baseline where significant low density or hemorrhagic regions would not be expected; the 0–40 threshold for applied only for region of visible infarction segmented by the deep learning algorithm. The mean HU density (D) of all voxels (after CSF and threshold-based exclusions) within each region (ischemic vs. normal) was calculated and NWU was defined as:


(1) $$\begin{array}{r} NWU=1-\frac{D_{ischemic}}{D_{normal}} \end{array}$$

We also calculated NWU using the standard 20–80 threshold for the infarct region and compared results to those obtained using our modified approach. The code for the manipulation of the infarct region and calculation of NWU is publicly available at: https://github.com/dharlabwustl/csfratio_nwu.

**Figure 1:**
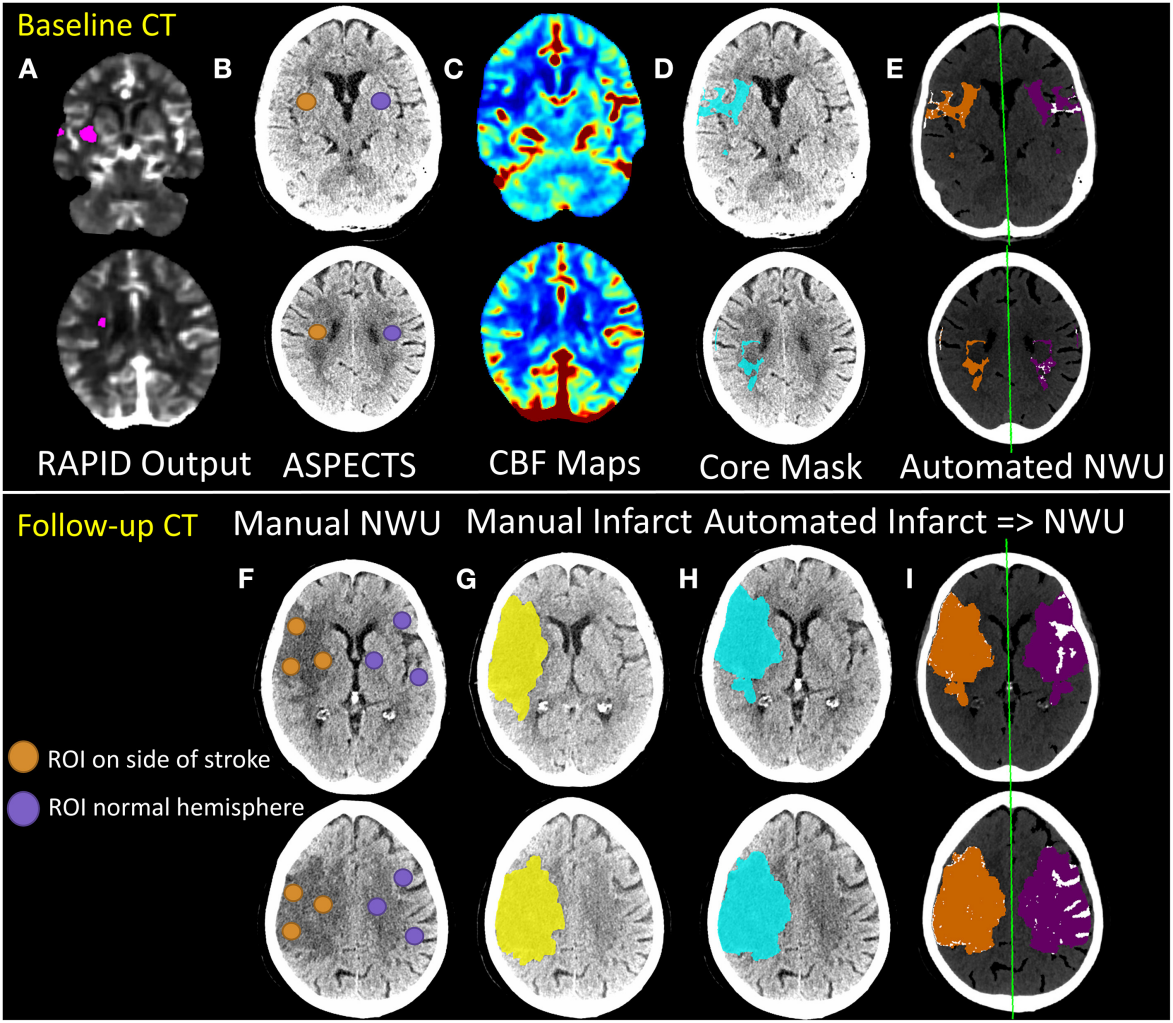
Outline of manual vs. automated techniques for estimation of net water uptake from baseline (top panels) and follow-up (bottom panel) CT scans of stroke patients. The RAPID core output **(A)** from CTP processing is used to determine the ASPECTS regions to be used for manual estimation of NWU from baseline NCCT **(B)**. Regions-of-interest (ROIs) are placed in these regions within the affected hemisphere (orange) and matched ROIs are placed in the contralateral hemisphere (purple). Manual NWU is calculated as one minus the ratio of mean densities of the two sets of ROIs. For automated measurement of NWU on baseline CTs, CBF maps **(C)** are generated from raw CTP data (as fully detailed in the Supplementary Methods and shown in Supplementary Figure 1). The core mask (defined by thresholding at CBF < 30% of normal) is then registered and overlaid onto the NCCT (blue region in **D**). This infarct region is then flipped across the midline (purple region; method fully outlined in the Supplementary Methods and shown in Supplementary Figure 2) to create a matching mirror region **(E)**. Automated NWU is calculated as one minus the mean densities of these two regions, after removing voxels of CSF (from separate CSF segmentation) or with HU density below 20 or above 80 from both regions (removed voxels shown in white). Lower panels show similar workflow for follow-up CTs (or baseline CTs with visible hypodensity). ROIs are placed within ASPECTS regions within the visible infarct and matching ROIs are placed in the contralateral hemisphere to calculate manual ROI-based NWU **(F)**. The infarct region is also manually segmented (yellow, **G**). A deep learning-algorithm is applied to automatically segment regions of hypodensity and generate an infarct mask (blue, **H**). Infarct regions are then flipped to create matching mirror ROIs (purple, **I**). Regions of CSF are then removed, as are voxels outside the thresholds (HU 0–40 for infarct, 20–80 for normal brain). Automated NWU is then calculated. In this example, manual NWU on baseline CT was 16.0 and automated NWU was 12.3. For follow-up CT, the manual infarct volume was 135 ml and the automated volume was 143 ml. The manual NWU was 29.8 using ASPECTS ROI-method, 25.4 using the whole manual infarct, compared with 25.0 for the fully automated NWU.

Automated NWU results were compared to a sampling based method that places circular regions of ~10-mm diameter within the infarct in up to 13 territories across two axial brain slices, corresponding to the ASPECTS regions as well as three more subcortical regions (selecting regions with visible infarct, while avoiding those with hemorrhage or CSF, as outlined in a prior paper for manual NWU measurement) (14). These same regions were then mirrored to the normal hemisphere and mean density of each set of ROIs were obtained and from this the manual NWU was calculated. For baseline NCCTs where clear infarction was not visible, the RAPID core images were used to guide manual ROI placement within ischemic tissue. If no acute infarction was visible on follow-up CTs based on the consensus of two independent raters, then NWU could not be obtained manually (and hence this scan was excluded). Manual NWU measurements were performed by two independent raters for all follow-up CTs and for a subset of the baseline CTs. The manual NWU used for comparison was the mean of the raters' measurements. In addition, for those 28 subjects with manual infarct segmentations performed, comparisons of manual volumes with automated infarct volumes as well as NWU values obtained using these manual vs. automated infarct masks were performed. Figure 1 outlines the major steps in obtaining manual vs. automated NWU measurements on baseline and follow-up CTs. Supplementary Figure 2 provides a more detailed, step-by-step outline of the automated image analysis workflow.

### Statistical Analysis

All analyses were performed in R (version 4.0.3, Foundation for Statistical Computing, Vienna, Austria). All measurements were first assessed for normality using inspection of their distributions and with Shapiro-Wilk's tests. We present means (with standard deviations) for normally distributed and medians (with interquartile ranges) for all non-normally distributed variables. We compared automated to manual measurements of NWU and infarct volume using intraclass correlation coefficients (ρ) with a two-way random effects model evaluating absolute agreement of raters, with the manual NWU as the ground-truth (ICC 2,1), using the package *psych* (29, 30). We constructed Bland-Altman plots of the difference between the two measures using the package *BlantAltmanLeh*. This allowed us to calculate the bias (mean difference) and limits of agreement (range within which 95% of differences in measurement lie).

## Results

**Figure 2:**
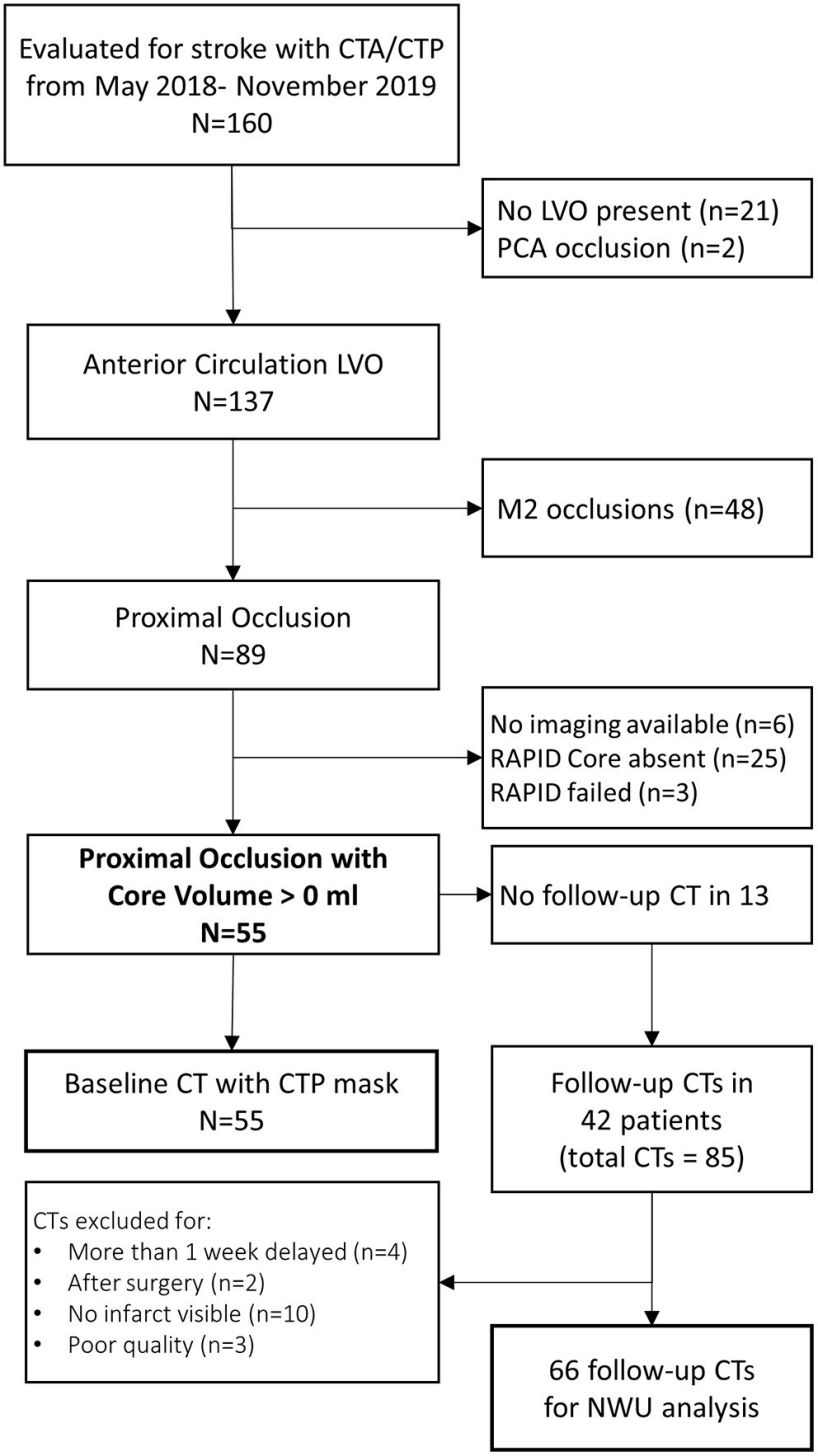
Flow of stroke patients assessed for eligibility for this imaging analysis.

We evaluated 160 patients who underwent acute stroke evaluation at our institution during the study period for eligibility. Of these 89 had LVO affecting the ICA or M1 segments and 55 had infarct core present on baseline imaging (see Figure 2). A description of this cohort is provided in Table 1. Mean NIHSS on presentation was 17 and 35 (64%) underwent thrombectomy (27 achieving mTICI 2b/3 reperfusion). Median time to baseline CT was 5 h (IQR 2–10). Median ASPECTS was 8 (IQR 6–10). Over one-third developed cerebral edema with midline shift (median of 3.7 mm, IQR 2.8–5.7) (time to scans).

**Table 1:** Characteristics of study cohort of 55 patients with large vessel occlusion stroke.

Age, years	74 (65–84)
Sex, female	27 (48%)
Race/ethnicity: white, non-Hispanic	45 (82%)
Hispanic	1 (2%)
African-American	9 (16%)
History of atrial fibrillation	24 (44%)
History of diabetes mellitus	14 (25%)
NIHSS, baseline	17 ± 6
NIHSS, 24-h	16 ± 10
Glucose (mg/dl)	121 (106–154)
Onset to baseline CT, hours	5 (2–10)
ASPECTS <7	15 (27%)
Treated with tPA	23 (42%)
Treated with Thrombectomy	35 (64%)
Reperfusion outcome: mTICI 0	3
mTICI 2a	4
mTICI 2b/2c	12
mTICI 3	15
Core volume (RAPID), ml	31 (14–65)
Penumbra volume (RAPID), ml	133 (94–175)
Midline shift	19 (35%)

### NWU on Baseline CTs

**Figure 3:**
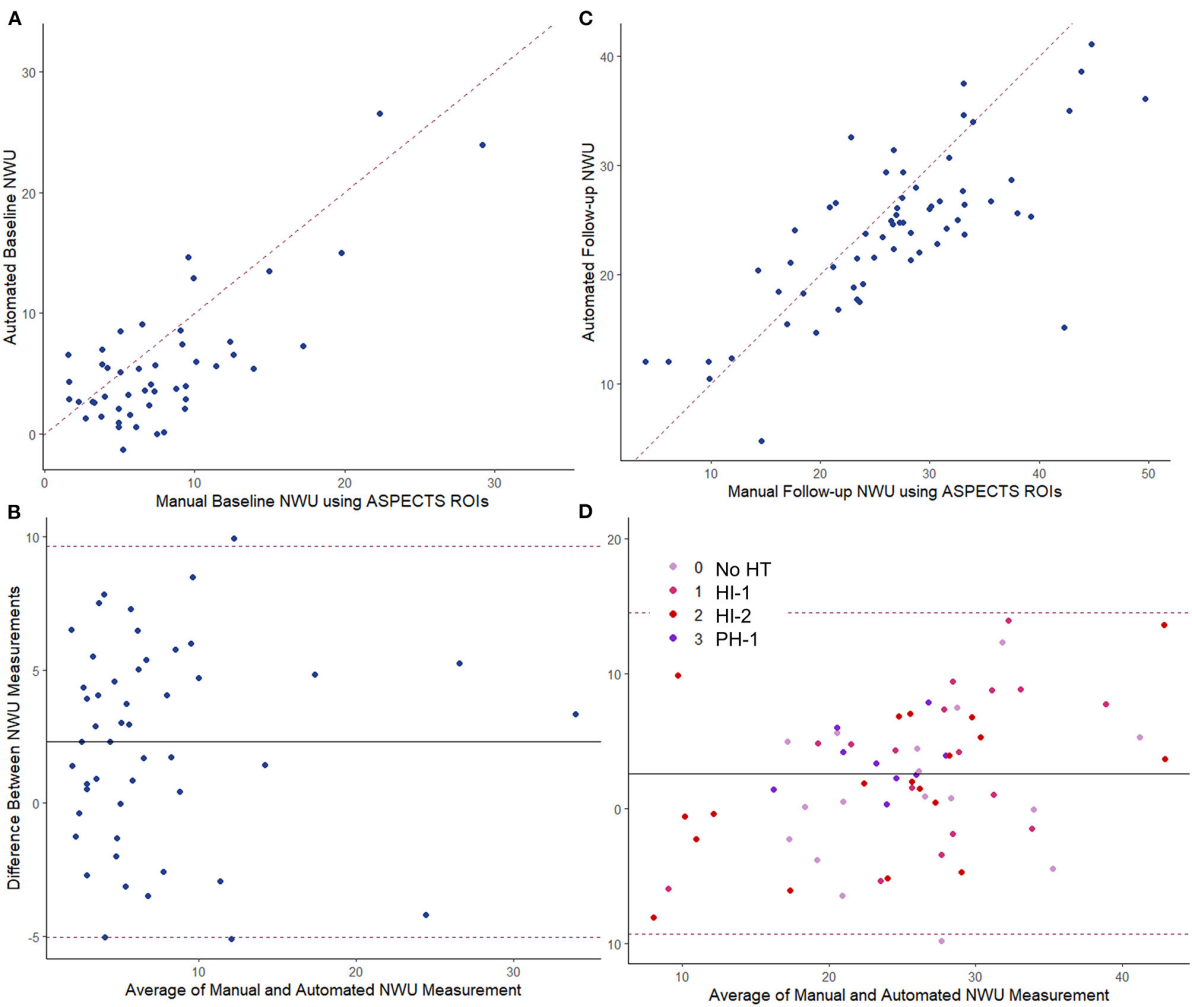
Comparing manual vs. fully automated measurements of NWU. Baseline CT: **(A)** intraclass correlation, ρ = 0.80; **(B)** Bland-Altman plot. Follow-up CT: **(C)** intraclass correlation, ρ = 0.68; **(D)** Bland-Altman plot with points colored by hemorrhage transformation (HT) type. The dashed line in the scatter plot represents the line of identify between measurements. The dashed lines in the Bland-Altman plots represent the limits of agreement. The solid line represents the mean difference in measurement (bias).

Baseline CTP images (median time from stroke onset of 4.3 h, IQR 2–10) were analyzed for core volume. Median core volume provided by RAPID software was 31 ml (IQR 14–65). Automated CTP processing allowed core masks to be extracted in 49 (89%) cases with a median volume of 36 ml (IQR 23–77). There was a strong intraclass correlation between the RAPID core volumes and our algorithm's volumes (ρ = 0.81, Table 2; Supplementary Figure 4). One case (with RAPID core of 4-ml) had no identifiable core on our processing and so no mask could be extracted. Two NCCTs had too much artifact to allow calculation of either manual or automated NWU. Baseline NWU using the manual ASPECTS ROI method was a median of 6.9 (IQR 4.2–10.0). The agreement of two manual raters for baseline NWU, tested in 22 cases, was good (ρ = 0.73, *p* = 1 × 10^−10^). In five cases with visible infarct on baseline CT, we used the automated infarct mask to define the infarct region; the automated CTP core masks were used to measure NWU in the remainder. There was strong agreement between manual and automated baseline NWU measurements (ρ = 0.80, *p* = 1 × 10^−16^) with minimal bias (Table 2; Figures 3A,B). The patch-based approach for NWU estimation exhibited modest concordance with manual methods (ρ = 0.63, *p* = 2 × 10^−8^) but with wider limits of agreement (−9.0 to 12.8, see Supplementary Figure 5).

**Table 2:** Comparison of manual and automated measures of core/infarct volume and net water uptake (NWU).

**Comparison**	**Manual value** **Median (IQR)**	**Automated value** **Median (IQR)**	**Mean difference** **Limits of agreement**	**Intraclass correlation** **(95% CI)**
Core volume: RAPID vs. automated CTP	31 (14–65) *N* = 55	36 (23–77) *N* = 49	−2.0 (−61.7 to 57.7)	0.81 (0.71–0.87)
Baseline NWU ASPECTS ROI vs. automated^†^	6.9 (4.2–9.9) *N* = 53	4.3 (2.6–7.3) *N* = 49	2.3 (−5.0 to 9.7)	0.80 (0.61–0.89)
Baseline NWU ASPECTS ROI vs. automated patch	7.0 (4.0–8.6) *N* = 50	5.5 (2.5–8.0) *N* = 50	1.9 (−9.0 to 12.8)	0.63 (0.47–0.75)
Follow-up infarct volume Manual vs. automated^†^	105 (39–140) *N* = 28	110 (39–194) *N* = 63	−12.1 (−60.9 to 36.7)	0.96 (0.92–0.97)
Follow-up NWU: Manual mask vs. automated^†^	25.3 (19.7–27.8) *N* = 28	24.6 (19.8–26.9) *N* = 63	0.6 (−2.4 to 3.6)	0.98 (0.96–0.98)
Follow-up NWU ASPECTS ROI vs. automated^†^	27.1 (21.9–31.7) *N* = 66	24.6 (19.8–26.9) *N* = 63	2.7 (−9.2 to 14.6)	0.68 (0.52–0.79)
All NWU values ASPECTS ROI vs. automated^†^	19.0 (7.3–28.1) *N* = 126	17.5 (5.5–25.3) *N* = 117	2.4 (−8.1 to 12.7)	0.88 (0.81–0.92)

*^†^Automated measurement using full-infarct mask (i.e., automated infarct segmentation or automated CTP core)*.

The automated NWU on baseline CT was negatively correlated with ASPECTS score (*r* = −0.60, *p* < 0.0001, Supplementary Figure 6), comparable with that observed for manual NWU (*r* = −0.59) but higher than for the patch-based NWU (*r* = −0.45). Automated NWU was higher in those who subsequently developed edema with midline shift (median 6.1 vs. 3.6, *p* < 0.0001). The automated NWU increased with longer time from stroke onset (beta = 0.12 per h, *p* = 0.0003), but the slope was significantly higher in those who subsequently developed edema with midline shift (*p* = 0.017 for interaction of edema with time, Supplementary Figure 7).

### NWU on Follow-Up CTs

There were a total of 85 follow-up CTs performed in 42 subjects. Of these, two were performed after hemicraniectomy and four scans were from more than 1 week after stroke. Of the remaining 79 scans, 69 exhibited visible regions of infarction. Three were too poor quality for NWU estimation, leaving 66 CTs for evaluation of NWU. Median time to follow-up CT was 60 h (IQR 35–116) with 27 (41%) being within 48 h of stroke onset. Hemorrhagic transformation was present in 48, including HI-1 in 18, HI-2 in 22, and PH-1 hematomas in 9 scans. The automated infarct segmentation algorithm correctly identified the region of infarction in all but two cases (97%). Manual delineation of infarct regions was performed in 28 scans with excellent correlation to the automated volumes (ρ = 0.96, see Supplementary Figure 8). The DSC for overlap of infarct segmentation (automated vs. manual) was a median of 0.83 (IQR 0.72–0.90) with a median automated infarct volume of 110-ml (IQR 39–194). There was no significant correlation between infarct volume and DSC (*r* = 0.26, *p* = 0.18) or time to scan and DSC (*r* = 0.32, *p* = 0.10).

**Figure 4:**
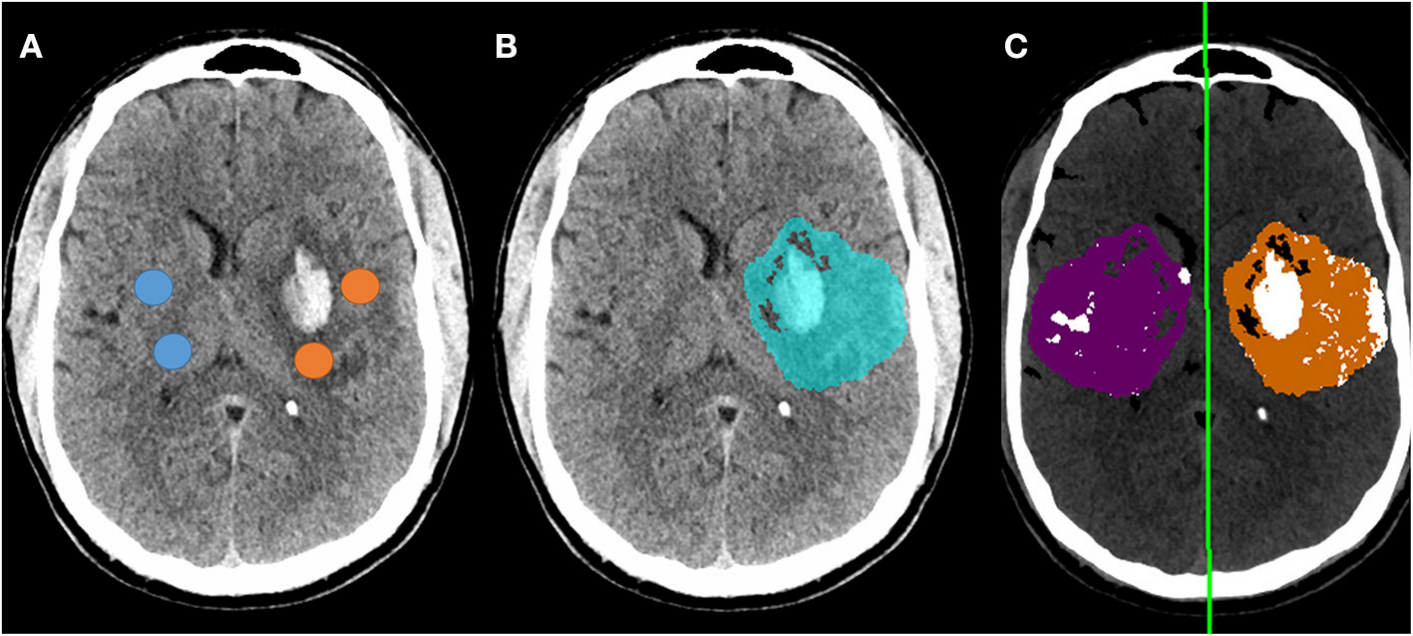
Example of follow-up CT in a patient who developed a focal parenchymal hematoma within the region of infarction. **(A)** Original non-contrast CT with regions-of-interest manually placed within the infarct (avoiding hemorrhage) and in contralateral matching regions (avoiding CSF); **(B)** Blue region indicates automated segmentation of infarct lesion; **(C)** Processing of follow-up CT to measure NWU using automated infarct mask, with removal of voxels representing CSF (white regions within purple normal mask) and voxels outside the range of 0-40 HU (removing most regions of hemorrhage). The manual NWU was 21.0 and the fully automated NWU was 21.6.

NWU was manually estimated on these 66 scans by two raters, using the ASPECTS ROI-based approach, with strong intraclass correlation between raters (ρ = 0.88, *p* = 3 × 10^−54^). Fully automated NWU was obtained by applying the automated infarct segmentation in 63 cases (two excluded for failure of infarct segmentation, one due to lack of voxel information in file header). The automated NWU (median 24.6, IQR 19.8–26.9) exhibited excellent agreement with NWU obtained from the manually drawn infarct masks (ρ = 0.98, Supplementary Figure 9). These values also correlated well with the manual ROI-based method (ρ = 0.68, Figure 3C). There was minimal bias (mean difference of 2.7) in NWU measurement, with no greater discrepancy in those with increasing severities of hemorrhagic transformation (Figure 3D). For example, in one case with focal hematoma, the manual NWU was 21.0 and the automated value was 21.6 (Figure 4).

NWU results using our modified thresholding (0–40 for the infarct region) were compared with the standard thresholding of 20–80. NWU was significantly lower with the original method (median 16 vs. 24.6) and underestimated the manually ascertained NWU (median 25.3). This was due to higher mean HU measured within the infarct region as a result of both exclusion of infarct voxels with density below 20 HU (i.e., regions of more severe edema) and inclusion of regions of hemorrhagic transformation within the infarct region. A review of voxels densities within infarct regions is shown in Supplementary Figure 10, highlighting the fact that 14% had densities below 20 HU and would have been excluded using the 20–80 thresholds. A comparison of the two thresholding methods for one representative example is shown in Supplementary Figure 1, where the modified approach yielded NWU of 24.7 while the standard threshold of 20–80 provided an estimate of 13.7 for NWU. The manual ROI-based method calculated by two raters was an average of 27.2. The overall correlation with manual NWU was lower (ρ = 0.43) compared with that obtained using the modified 0–40 approach (ρ = 0.66). Furthermore, the automated 20–80 method under-estimated the manual measurement (mean difference of −11.3), with wider limits of agreement, ranging from +3 to −26.

We then combined all automated NWU measurements together (total of 117 baseline and follow-up measurements): there was a strong correlation of automated to manual NWU across time points (ρ = 0.88, *p* < 0.0001) with minimal bias (Supplementary Figure 11). There was no difference in the accuracy of NWU (automated vs. manual discrepancy) based on time to scan, infarct volume, or degree of hemorrhagic transformation.

## Discussion

Quantifying the evolution and severity of cerebral edema, using routine CT imaging, presents the promise of accelerating research into clinical and biologic factors mediating this critical source of secondary injury (21). The application of NWU in small LVO cohorts has already led to several interesting observations: for example, those with worse collateral scores exhibited greater early edema progression (9). Hyperglycemia was also associated with enhanced edema formation (31). Estimation of NWU, which represents the proportion of stroke lesion that constituted of excess water, allows the separation of primary infarct from secondary edema within an infarct-related hypodensity on subacute CT (32). Not only does this allow delineation of edema severity but this adjusted infarct volume correlates well with final infarct volume, an important stroke outcome measure. Furthermore, it is likely that the product of infarct volume and NWU provides a meaningful volumetric measure of water accumulation, termed total edema volume (33). However, despite this promise, measurement of NWU in these previous studies was performed manually and hence was time-consuming, requiring investigators to outline the region of infarction across multiple CT slices, mirror this region to the normal hemisphere, and then measure the density in both, applying pixel-based intensity thresholds to try to exclude CSF regions that might contaminate the measurement.

We now present a substantial advance to facilitate scalable NWU measurement: that is, an automated approach that accurately quantifies NWU from both baseline and follow-up CTs. This was built upon two new algorithms to extract regions of infarction: one a deep learning model that was able to segment the region of infarct-related hypodensity from follow-up CTs and a second that extracted CBF-based core masks from baseline CTP images. While the latter is qualitatively similar to the automated processing of core volumes provided by several commercial software packages (34), it is performed at native resolution and provides voxel-by-voxel maps for research applications. In comparison, output from RAPID software is not provided in native resolution and cannot be easily incorporated on a voxel-by-voxel level for analysis.

Segmentation of infarct regions from follow-up CTs in the first days after stroke is not a trivial task, as early hypodensity can be subtle and challenging to delineate even for experienced human raters. Simple thresholding of CT images for infarct is not accurate as infarcts overlap in density with CSF and other brain structures. An approach utilized in the MR CLEAN cohort applied an intensity-based region-growing approach that begins with a manual seed and also excludes neighboring ventricular CSF regions that would be misinterpreted as infarction. This multi-stage process exhibited similar accuracy to that achieved with our algorithm (i.e., both had correlation of automated to manual infarct volumes of 0.98) (35). Two recent deep learning-based approaches have been proposed for this task: one employed a combination of three patch-based convolutional neural networks to identify infarct regions from follow-up scans in the HERMES collaborative (25). This method (trained on 630 scans, tested on 396) resulted in a lower DSC for infarct segmentation (0.57 vs. 0.83) and lower ICC (0.88 vs. 0.96) for infarct volumes measurement than seen with our algorithm, although we cannot directly compare without head-to-head testing on a single cohort, given differences in infarct types (for example, the median infarct volume in our series was 110 vs. 48-ml in the other study). The performance for larger, more hypodense infarcts was higher, with DSC of 0.78 and ICC of 0.98, comparable to our findings. The second study employed a generative adversarial network (GAN) to enhance the U-Net architecture (36). This was trained on 60 scans and tested on 60 others, with DSC of 0.71 and correlation to manual volumes of 0.93. The advantage of this automated method is that it does not require human input and so can segment large numbers of scans within the context of the broader automated NWU algorithm. Given a few outliers where segmentation failed (<5% of cases), we still recommend manual review of the infarct/NWU results. However, there is likely some leeway as long as the segmented infarct region overlaps broadly with the infarct for estimation of the mean infarct density.

These advances allowed us to measure NWU on both baseline and follow-up CTs, while most studies have focused on a single time point. We applied these infarct masks, along with technical developments to flip the region along the registered midline, to obtain the ratio of densities between these two regions (i.e., NWU). In doing so, we modified the prior thresholds used for NWU calculation. As in prior studies (25), we noted that a significant portion of infarcted tissue had HU below 20 and so excluding voxels with HU < 20 would lead to under-estimation of the actual infarct density and therefore NWU (as shown in comparing the results obtained from the two thresholds) (25). One reason for thresholding at 20 HU is to avoid contamination of the regions by low-density CSF, an issue in prior studies; we applied our well-established CSF segmentation algorithm to subtract these non-brain regions and avoid such contamination.

Presence of hemorrhagic transformation within the infarct has also confounded measurements of NWU, as voxels with hemorrhage would have high HU and would lead to significant underestimation of the actual difference in densities between infarct and normal brain. This bias has often led to those scans with significant HT being excluded from NWU studies. This is a major drawback, as HT occurs in 20–40% of LVO patients (37), limiting the number of patients who can be analyzed and generalizability of findings. A recent study of edema measurement after thrombectomy found that presence of hemorrhage and/or contrast staining often contaminated measurement of NWU, leading to under-estimation and even some negative NWU values; this issue compromised the value of NWU in comparison to volumetric edema biomarkers (28). We did not exclude those with HT but applied an upper threshold of 40 H; this removed regions of significant HT (as shown in Figure 4) and allowed us to measure NWU from all scans (including those with variable degrees of hemorrhage) without any noticeable increase in error. However, our cohort did not include any patients with very large PH-2 hemorrhages; in such cases where the hemorrhage encompasses the entire region of infarction, NWU could not be calculated by any method. We demonstrate that the NWU obtained from our automated algorithm accurately reflected those obtained using manually-drawn infarct regions and those using an established ASPECTS ROI-based method for both baseline and follow-up CTs (14). There was only a small bias, with automated NWU providing lower values (by ~2%) than manual NWU; this might actually be because the manual method involves sampling select regions within the infarct (or core) and thereby focuses on more obvious hypodense regions where NWU is higher (compared with our full-infarct method, which measures edema using the full range of densities within the infarct) (8).

There have been other recent attempts to operationalize and simplify measurement of NWU. Notably, all have focused on estimation of NWU from baseline CTs only and most require manual region selection or other input/review. One approach applied an automated core region (similar to our CTP-based method) but derived from commercial software (38); it was unclear how this region was transferred to the NCCT as alignment of CTP source images with NCCT is not trivial, but likely involved manual inspection and alignment. Another study manually placed a standard large ROI within the MCA territory (on a single slice) to estimate where ischemia might be seen and avoid the need to use CTP to locate the exact core region (39). Baseline NWU was estimated from the density of this region vs. a manually translated contralateral mirror region, but was not compared to a gold-standard measurement. Only one automated method has been proposed: using commercial software (syngo.via from Siemens) to measure the density within affected ASPECTS regions on baseline CT (40). This technique approximates our manual ASPECTS-based sampling method but has the advantage of not requiring CTP for NWU measurement and being relatively automated (manual inspection of regions was required to check accuracy). We were not able to compare our automated method to this automated ASPECTS-NWU approach as we did not have access to the commercial software, but did use a comparable manual ASPECTS approach as our ground-truth for NWU.

Finally, a fourth study applied standardized regions (patches) within the MCA territory to obtain relative density and estimate NWU without requiring CTP to generate core masks (41). However, these regions had to be manually selected to avoid regions of CSF and old infarcts and would tend to under-estimate NWU as it inevitably includes non-ischemic regions. In that study, this patch-based method was compared to a full-infarct approach (based on region of infarction on follow-up CT, manually outlined) and found limits of agreement from −9 to +10, similar to what we found (−5 to +9.7). For comparison within our own dataset, we implemented a fully automated version of this patch-based sampling method to measure NWU from the baseline CTs and showed that it exhibited moderate agreement with manual ASPECTS region-based measurements and with our automated approach. This is likely because this method places regions within the MCA territory, presuming that these will capture the ischemic tissue with early edema. As might be expected from a sampling method that estimates early edema, this approach exhibited wider limits of agreement compared to our full-infarct/core based method. Nonetheless, it is an attractive complementary or back-up option if CTP is not available to define core and the infarct is not visible.

These studies also confirmed, as we did, that baseline NWU was higher in those who subsequently developed midline shift and/or malignant edema. However, none of these studies incorporated automated assessment of NWU from follow-up CTs. Our method is therefore more comprehensive in allowing measurement of NWU from serial CTs at various time points in a fully automated manner (without manual inspection, flipping of masks, etc.). We believe that this approach will empower large cohort studies and clinical trials that seek to understand the dynamic evolution of edema after stroke (14).

There remains limitations to applying NWU to study edema, regardless of measurement approach. As stated above, NWU cannot be used to estimate edema in the presence of extensive parenchymal hematoma. In these cases, water accumulation is unlikely the main contributor to midline shift and deterioration (37). Instead, efforts at measuring hematoma volume may allow quantification of secondary injury better than NWU (42). Concomitant CTP with measurable core volume is generally required to facilitate measurement of NWU from baseline CTs unless infarct is clearly visible (as it was in only five of our 55 cases); this limits its applicability to studies of early edema. Application of ASPECTS-based approaches may obviate the need for CTP but rely on presence of early ischemic changes and would not be possible in those with “normal” appearing baseline CTs (i.e., ASPECTS = 10). Similarly, NWU cannot be assessed from early follow-up CTs before infarct hypodensity is clearly visible. There were several CTs in our cohort where either no infarct was visible or it was too subtle for segmentation. We have recently demonstrated how volumetric CSF-based biomarkers of evolving edema can be measured and reflect edema formation within the first 12–24 h after stroke (23). It is possible that these two biomarkers capture different aspects of edema formation, one densitometric and other volumetric. The current study provides technical validation of our approach but was performed in a pilot cohort too small to analyze clinical outcomes. It also requires external validation in an independent cohort to ensure generalizability. We now plan on evaluating both density and volume ratios (i.e., NWU and CSF ratio) in larger cohorts using these automated algorithms so that we can understand the relation of these biomarkers to one another and to relevant edema outcomes.

## Data Availability Statement

The raw data supporting the conclusions of this article will be made available by the authors, without undue reservation.

## Ethics Statement

The studies involving human participants were reviewed and approved by Human Research Protection Office, Washington University in St. Louis. Written informed consent for participation was not required for this study in accordance with the national legislation and the institutional requirements.

## Author Contributions

RD contributed to the conception and design of the study, performed statistical analyses, and wrote the original draft. AK, YC, AA, ZV, and JD contributed to the technical development and analyses and as well as contributing and/or editing key sections of the manuscript. AC, AH, GC, KB, and LH contributed to the acquisition of data for the study. J-ML contributed to the conception of the study and provided critical revisions to the manuscript. All authors contributed to the manuscript revision and have read and approved the submitted version.

## Funding

J-ML received funding from NIH (R01NS085419 and U24NS107230), RD received funding from NIH (K23NS099440), LH received funding from NIH (K23NS099487), and JD was supported by the 2018-2020 ASNR/RSNA Scholar Award (RR1817) and Mallinckrodt Institute of Radiology.

## Conflict of Interest

The authors declare that the research was conducted in the absence of any commercial or financial relationships that could be construed as a potential conflict of interest.

## Publisher's Note

All claims expressed in this article are solely those of the authors and do not necessarily represent those of their affiliated organizations, or those of the publisher, the editors and the reviewers. Any product that may be evaluated in this article, or claim that may be made by its manufacturer, is not guaranteed or endorsed by the publisher.
