# Supplementary material for: Automated Measurement of Net Water Uptake From Baseline and Follow-Up CTs in Patients With Large Vessel Occlusion Stroke
Source: Front Neurol. 2022 Jun 27;13:898728. doi: 10.3389/fneur.2022.898728 (PMC9271791; doi:10.3389/fneur.2022.898728)
Supplement: Supplementary file 1 [file Data_Sheet_1.pdf]

Supplemental Methods

Detailed workflow outlining steps in the automated measurement of NWU

The target axial non-contrast head CT (NCCT, A in Suppl. Figure 2) was converted to NIfTI format (1). To isolate the head region from background, levelset segmentation was performed using the Computational Morphometry Toolkit (CMTK; https://www.nitrc.org/projects/cmtk/). Automatic skull-stripping was performed with the brain extraction toolkit (BET) within FSL (B in the Figure) (2). The skull-stripped CT images were normalized by first subtracting the mean HU value within the brain and then dividing by two standard deviations so that most of the brain voxels will have normalized values within the range [-1,1].

A CT template (C in the Figure), developed by Rorden et al. (3) was resized to 512 x 512 x 53 voxels using a linear interpolator (SimpleITK), to create a template CT for this study (4). A 3D mask of midlines bisecting all the slices of the template was then synthesized (D).

This template CT was then registered to the target CT using FLIRT from FSL (5, 6). 3D registration with 12 degrees of freedom using a cost function of correlation ratio and a tri-linear interpolation was applied (E). The calculated transformation matrix was then applied on the template midline mask. The transformed midline points were further smoothed with RANSAC linear regressor fitted on the midline points for each slice. The RANSAC regressor had a random state of 0, maximum number of trials of 1000, a residual threshold of 1, and used all the points of the midline in each slice. The smoothed midline was then applied to the target CT (F).

Cerebrospinal fluid (CSF) regions within the target brain (after skull-stripping and normalization, as described above) were segmented using an established and validated deep learning approach. The CSF mask was superimposed onto the target CT (G) and was further divided into left and right hemispheric CSF based on the projected ideal midline (H). A linear algebra approach was used to determine the side of each voxel, using the equation:

$$d = (x - x_1)(y_2 - y_1) - (y - y_1)(x_2 - x_1)$$

where (x_1,y_1) and (x_2,y_2) are two points on the midline , and (x,y) is the 2D coordinate of a voxel in the CSF mask. If $d < 0$ then the voxel lies on the left side of the line, and if $d > 0$ then it lies on the right side. If $d = 0$ then the voxel lies exactly on the midline; such voxels were discarded.

The infarct mask (obtained as described below) was then applied to the target CT (image I in the Figure) and flipped to obtain the homologous regions in the contralateral non-infarcted hemisphere, as follows (using the OpenCV image processing library) (7): (i) the infarct mask, on each slice, was first translated so that the midpoint of the midline mask (shown in yellow) was aligned to the center of the image; ii) the resulting image was then rotated to make the midline parallel to the y-axis of the image; if any portion of the infarct mask crossed the midline, only the larger portion in one hemisphere was considered further (iii) this infarct mask then then flipped around the midline using reflection transformation to create a mirrored region in the normal hemisphere; (iv) the rotation and translation of the image was then reversed to bring the image back into its original orientation, by applying inverse transformations. This resulted in both

infarct and normal region masks in the original CT orientation. Both masks were then superimposed onto the target NCCT image (J).

Finally, the voxels to be used for calculation of infarct and normal region density were selected. First, voxels within each region that were segmented as CSF were removed (K). Then a threshold was applied: voxels with densities outside the range 20-80 were removed, based on prior approaches (image M) (8). For the infarct region, a modified threshold of 0-40 was applied instead, meaning that only voxels of very low density (HU < 0) or densities above that of normal brain (HU > 40) would be removed (image L). The mean density of all remaining voxels were then calculated for each region. Net water uptake (NWU) was calculated as one minus the ratio of densities within the infarcted versus normal region.

<u>Infarct Mask Segmentation</u>

1. CT perfusion processing for core masks (baseline CTP only)

CTP raw data was analyzed using in-house software written in MATLAB (MathWorks, Natick, MA) (9). This applied singular value decomposition deconvolution methodology (10-12). Unlike RAPID which down-samples image data in-plane by 2x, this algorithm analyzed data in native resolution. It applies rigid body motion correction, skull stripping using morphological image processing, brain connectivity identification, and automated extraction of the arterial input function (AIF) and venous outflow (aka tissue residual function, trf). It performs deconvolution to obtain tissue-response functions and extracts maps of T_{max}, MTT, CBV, and CBF for all slices. The region of core was extracted as follows: normal brain regions were defined as those with Tmax below four seconds. Mean CBF was calculated in these regions. Then core was defined as regions where voxels had CBF below 30% of this standardized normal flow. A mask of the voxels in the core region were then transferred to the NWU workflow for analysis. The workflow is shown in Supplemental Figure 1.

2. Segmentation of infarct masks (follow-up CTs and baseline CTs with visible hypodensity)

One investigator manually delineated visible regions of infarction on 335 follow-up NCCTs from a prior cohort of stroke patients from three institutions (13). The principal study investigator reviewed all masks for accuracy. These infarct masks were used to train a deep-learning based segmentation model, based on the fully convolutional U-Net architecture (14). The network had four layers in both the contracting and expanding paths (see diagram below). After each contraction layer, the feature numbers were doubled but size was halved, while features were manipulated in the opposite way in the expanding path. There are skip connections between the contracting and expanding paths at the same depth, to facilitate information flow within the network. The model was pre-trained to segment CSF from stroke CTs, as previously described (15). Training for infarct lesion segmentation was optimized by minimizing the binary cross-entropy between the network output and manual ground-truth, using an Adams optimizer with learning rate 0.0001. Dropout rate was set at 25%. Approximately ten percent (31) masks were used for internal validation to optimize hyperparameters.

The final output is a infarct segmentation probability map with voxels corresponding to the original NCCT. We defined infarct regions as those with probabilities ≥ 0.50. This region was further refined through region growing which evaluates if each voxel's neighbor was also within the infarct mask. This results in multiple connected regions in three dimensions, representing parts of the proposed infarct mask that are connected. Only the single connected region with the largest volume was then considered as the final infarct for analysis and other smaller regions were removed.

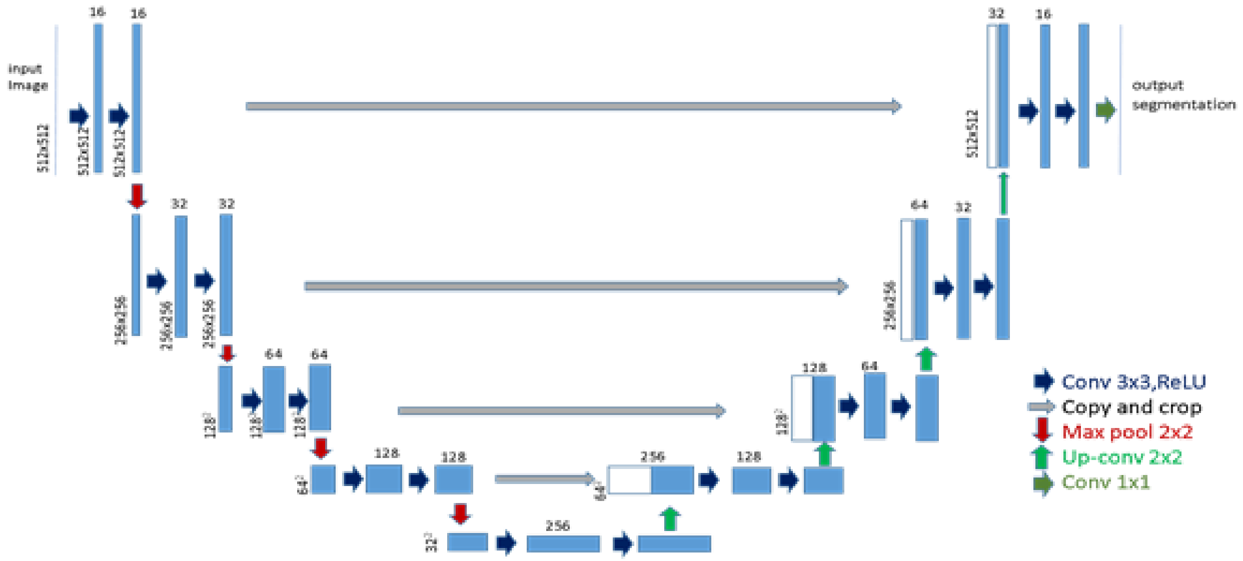

Supplemental Figures

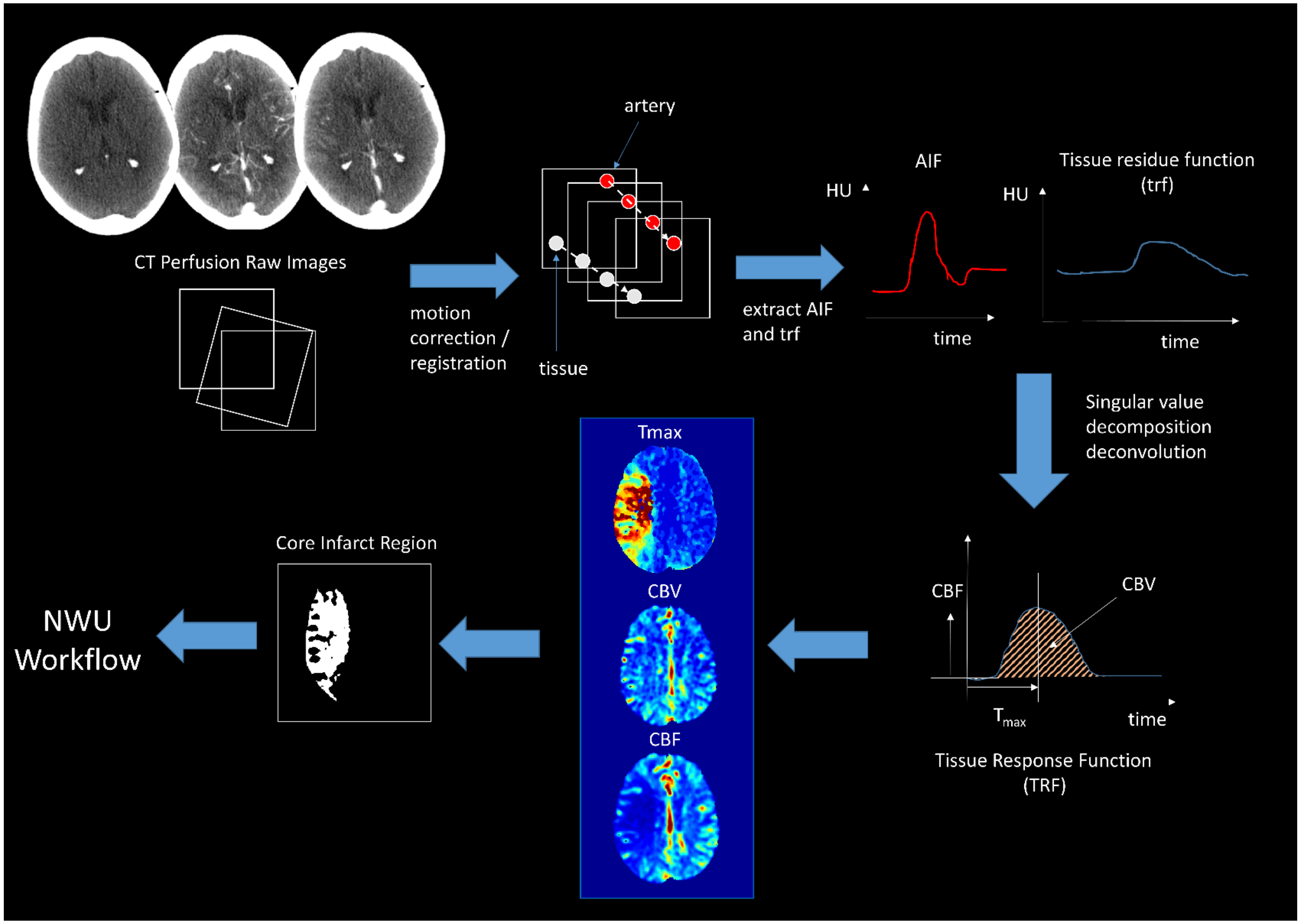

Supplemental Figure 1. Workflow for automated processing of CT perfusion images to obtain core infarct mask (as outlined in Supplemental Methods). The CTP images undergo motion correction and extraction of arterial input function (AIF) and tissue residue functions (trf). Deconvolution is then applied to obtain image maps of Tmax, CBV, and CBF. The core mask is obtained by thresholding the CBF map at 30% of the normal value, obtained by averaging CBF within regions with Tmax < 4 seconds.

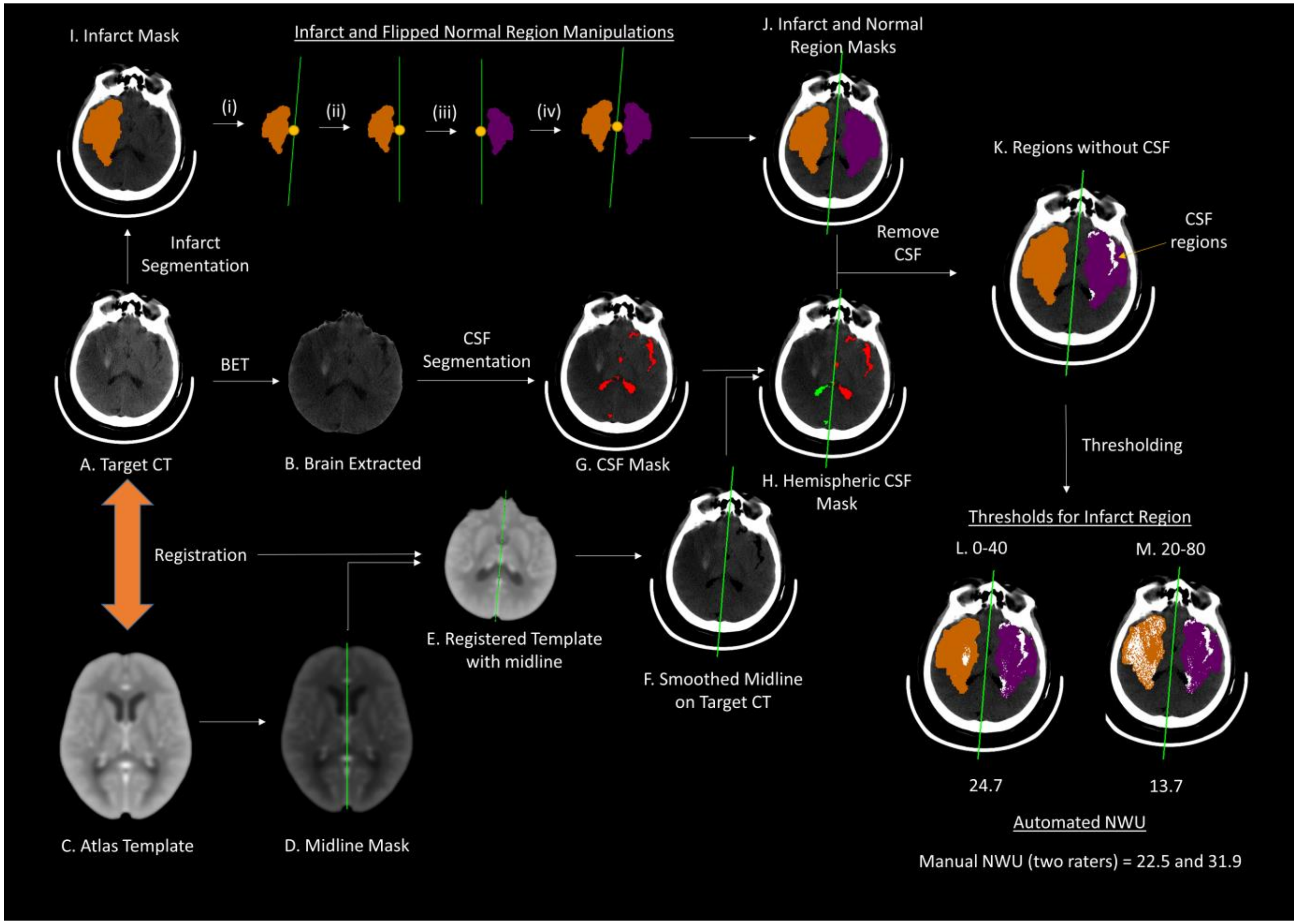

Supplemental Figure 2. Detailed workflow with steps for automated processing of CT images to obtain NWU (as outlined in Supplemental Methods). The original NCCT is shown as (A) with workflow that includes CSF (G) and infarct (I) segmentation. The infarct mask is flipped to obtain a mirrored normal region (J). NWU is calculated as the ratio of densities of the two regions after removal of CSF (K) and voxels with HU outside of thresholds (0-40 for infarct in L vs. 20-80 in M), resulting in NWU of 24.7 vs. 13.7 with the two thresholds. Two raters independently measured NWU on the same target CT using an ASPECTS-based method, obtaining values of 22.5 and 31.9.

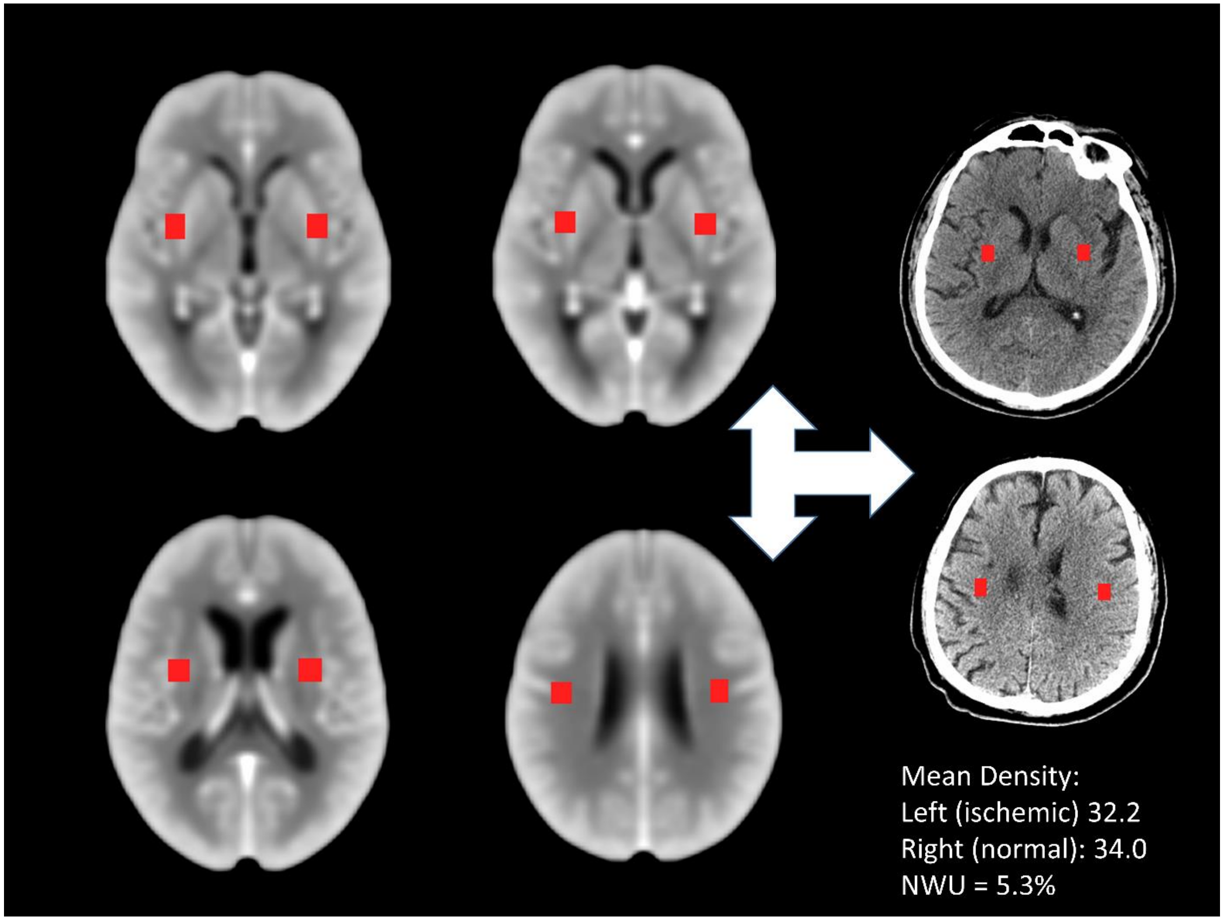

Supplemental Figure 3. Method for measuring patch-based NWU. Four patches in each hemisphere are translated from a brain atlas to each patient's baseline NCCT using linear registration. The mean density of these four regions is calculated and NWU obtained as one minus the ratio of the density in the affected vs. unaffected side (based on clinical and follow-up data). In the case shown, patch-NWU was calculated as 5.3%.

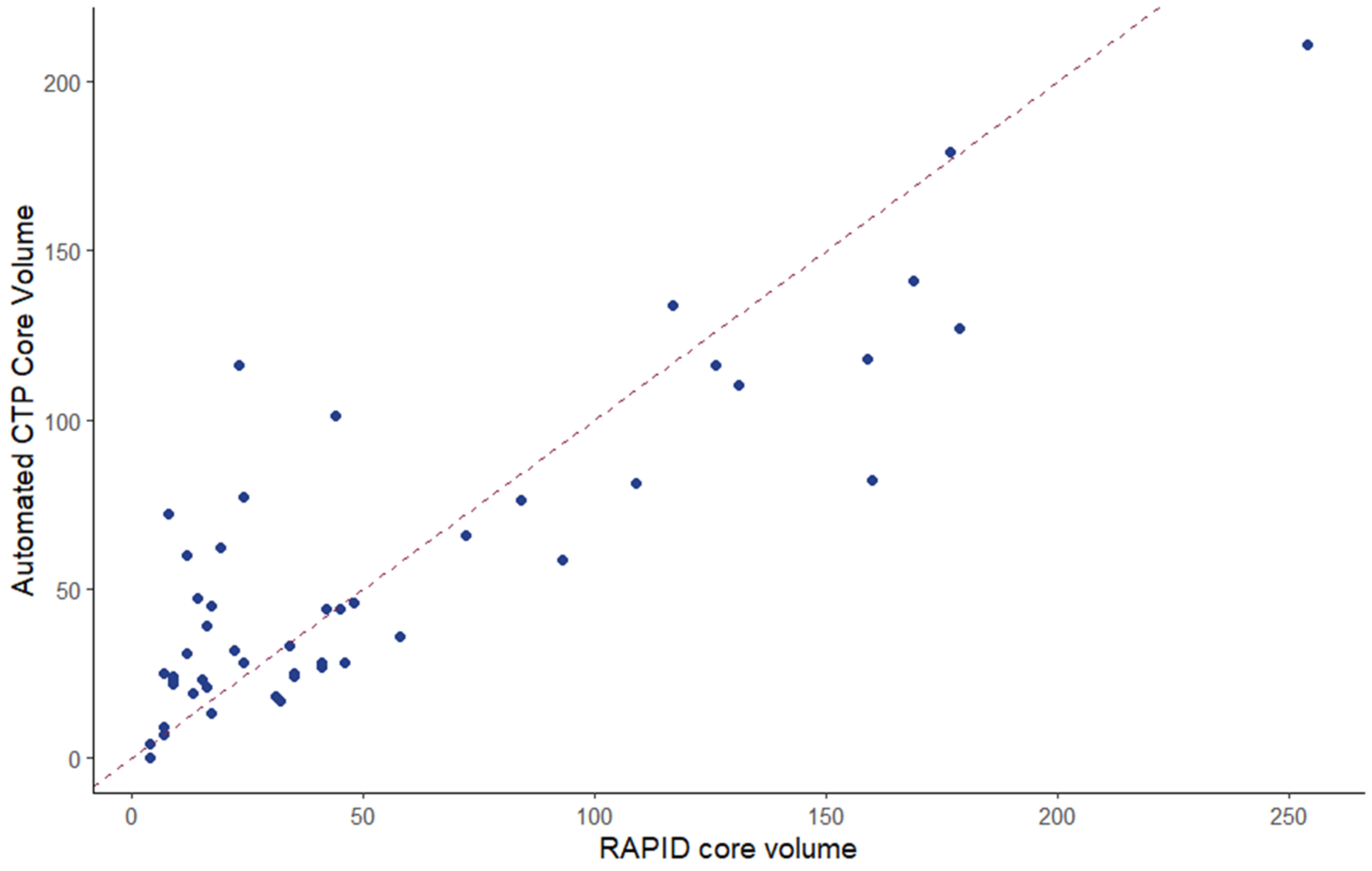

Supplemental Figure 4. Correlation between core volumes provided by RAPID software and the in-house automated CTP algorithm (intraclass correlation, ρ =0.81; dashed line represents line of identify).

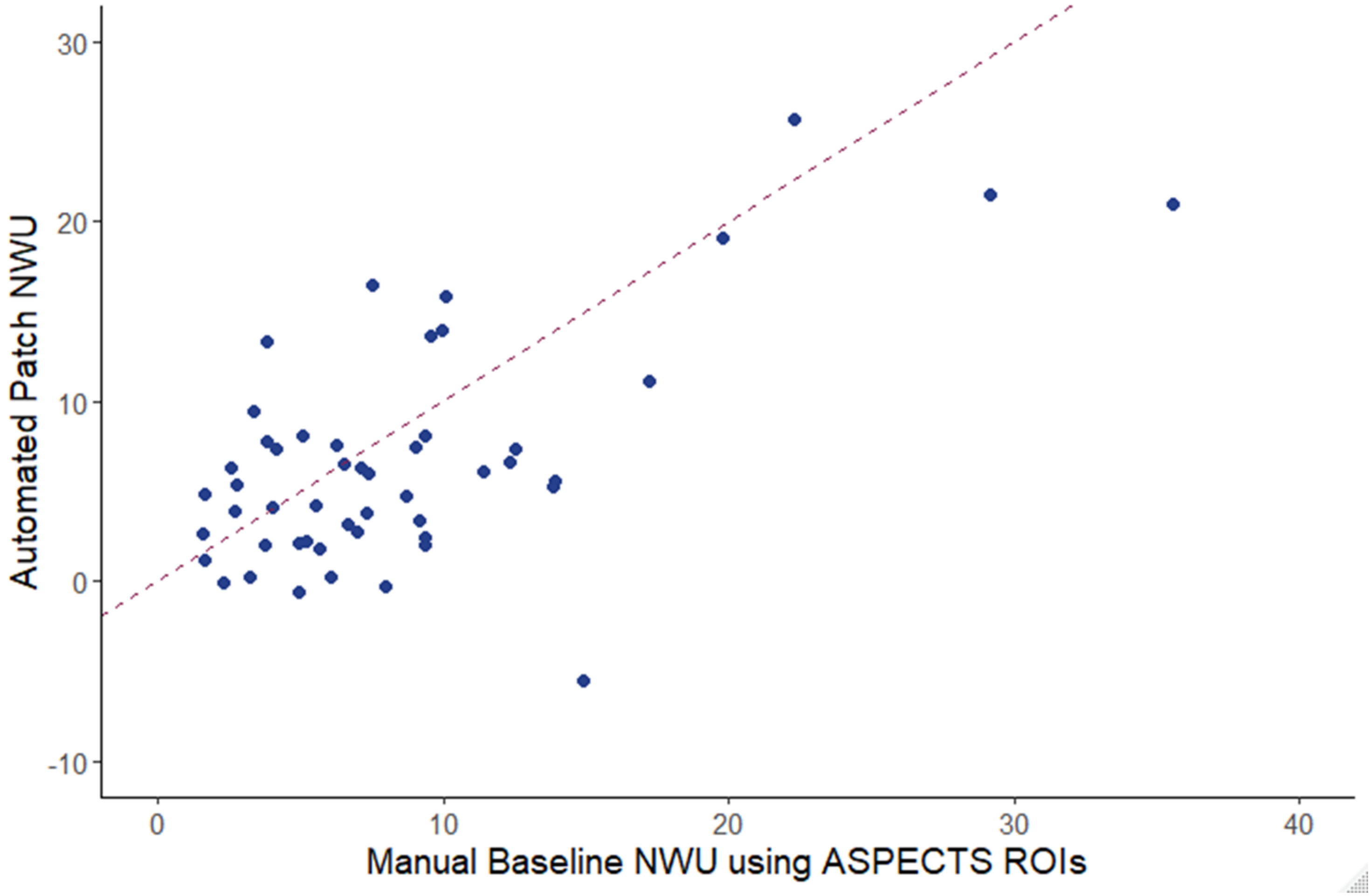

Supplemental Figure 5. Correlation between NWU values obtained using manual ASPECTS-based sampling within infarct core on baseline CT and automated patch-based method in 50 baseline CTs (ρ = 0.63; dashed line represents line of identify).

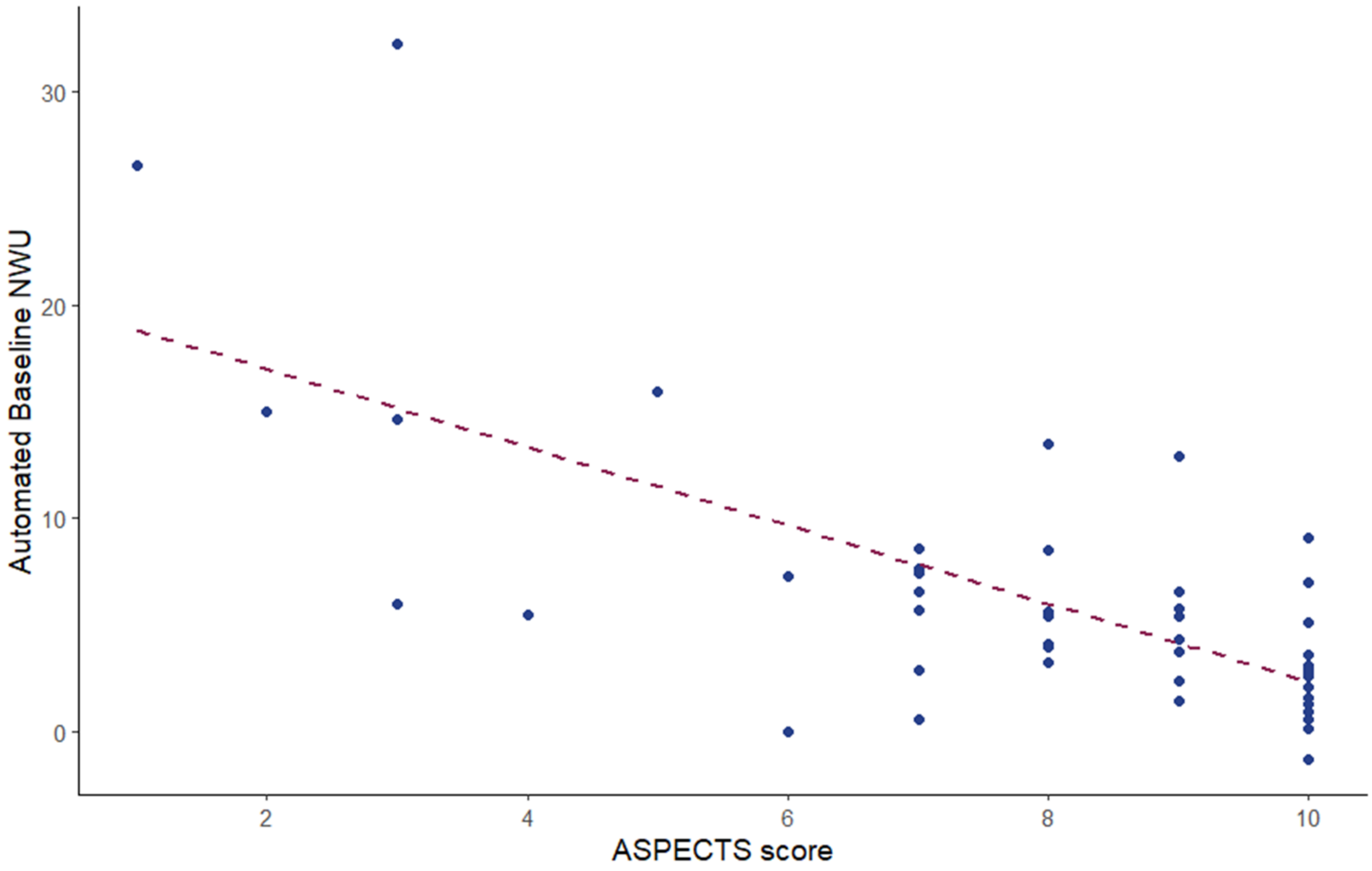

Supplemental Figure 6. Relationship of baseline automated NWU measurement to ASPECTS on baseline CT (r=-0.60 using Spearman rank correlation, p=0.0001).

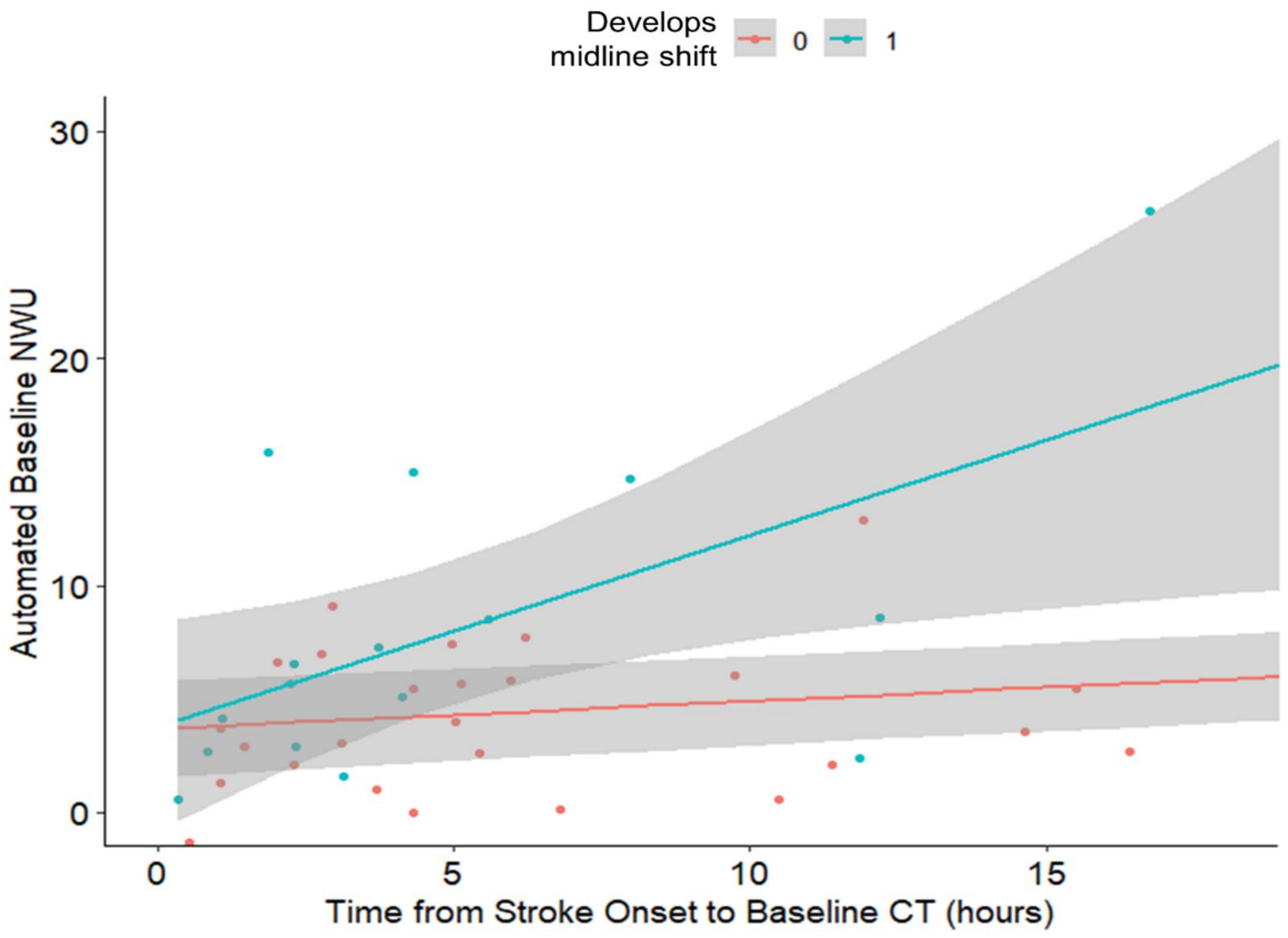

Supplemental Figure 7. Automated NWU measured on baseline CTs within 24 hours of stroke onset increases more with increasing time from onset in those who subsequently develop midline shift (blue) compared with those who do not (red). Trend lines represent results of linear regression for each group, with 95% confidence intervals.

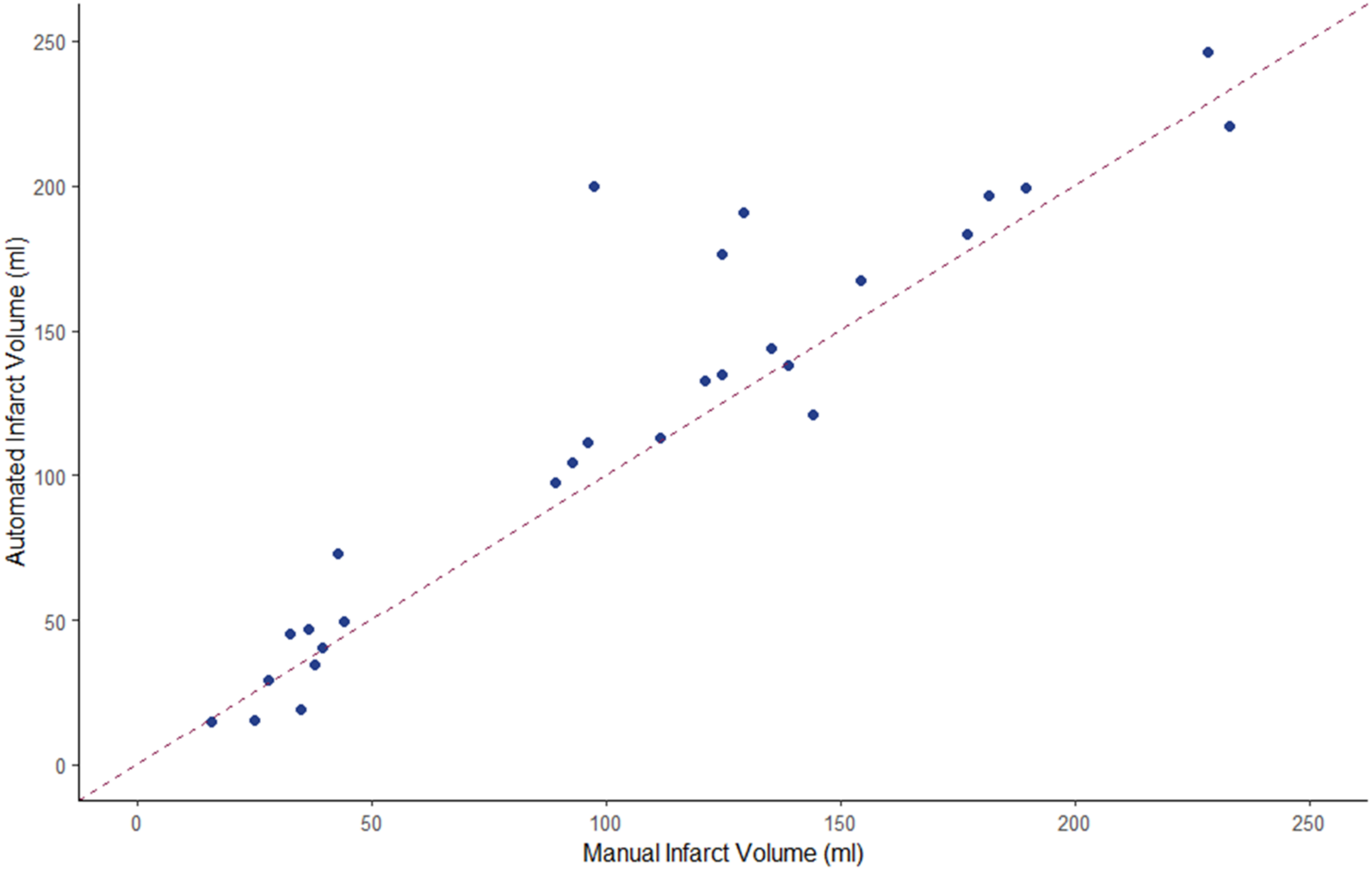

Supplemental Figure 8. Correlation of automated to manually segmented infarct lesion volumes in 28 follow-up CTs with visible infarcts (ρ=0.96; dashed line represents line of identify).

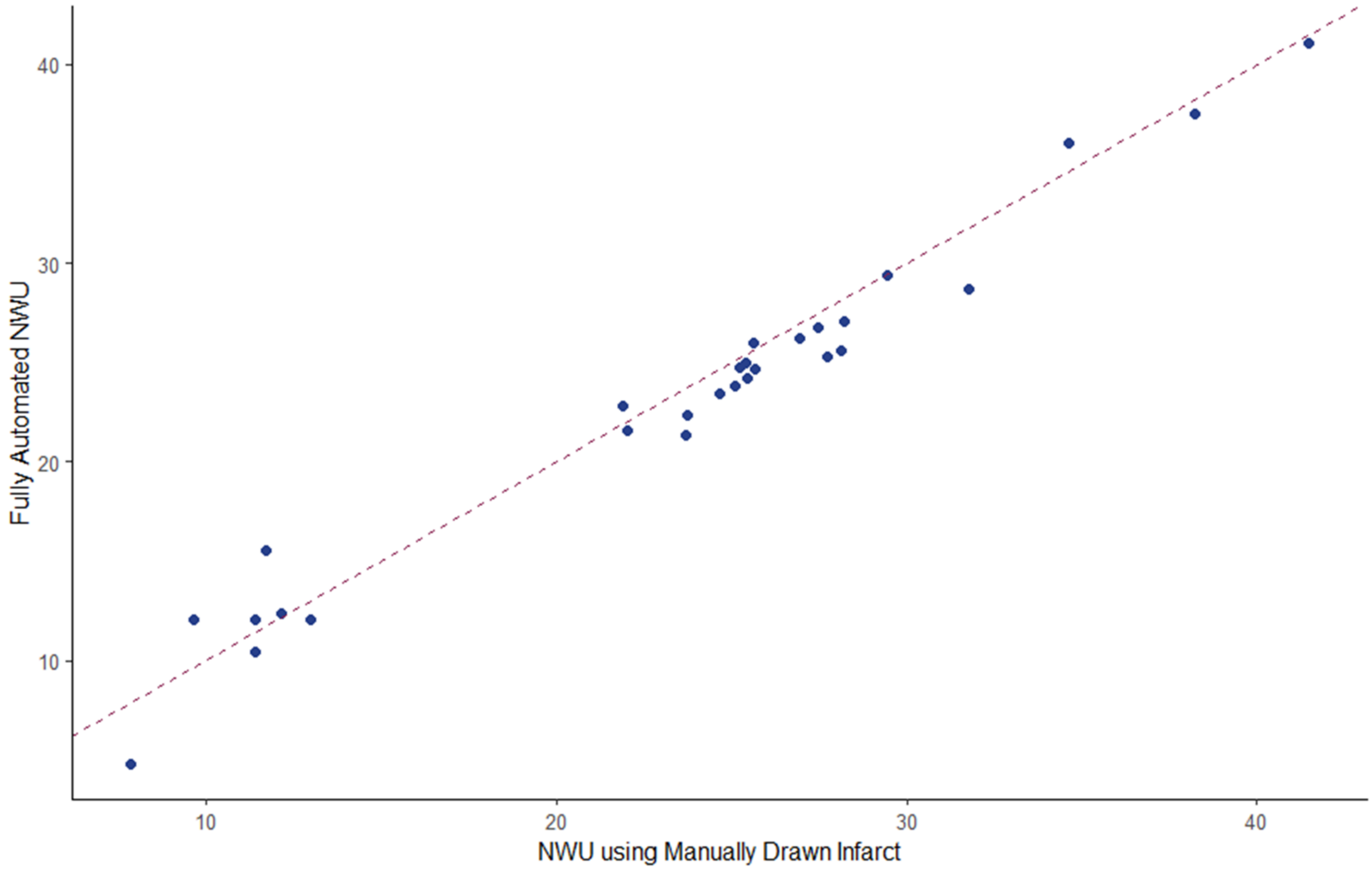

Supplemental Figure 9. Correlation of NWU obtained using manually delineated infarct regions to fully automated NWU using automated infarct masks (ρ=0.98; dashed line represents line of identify).

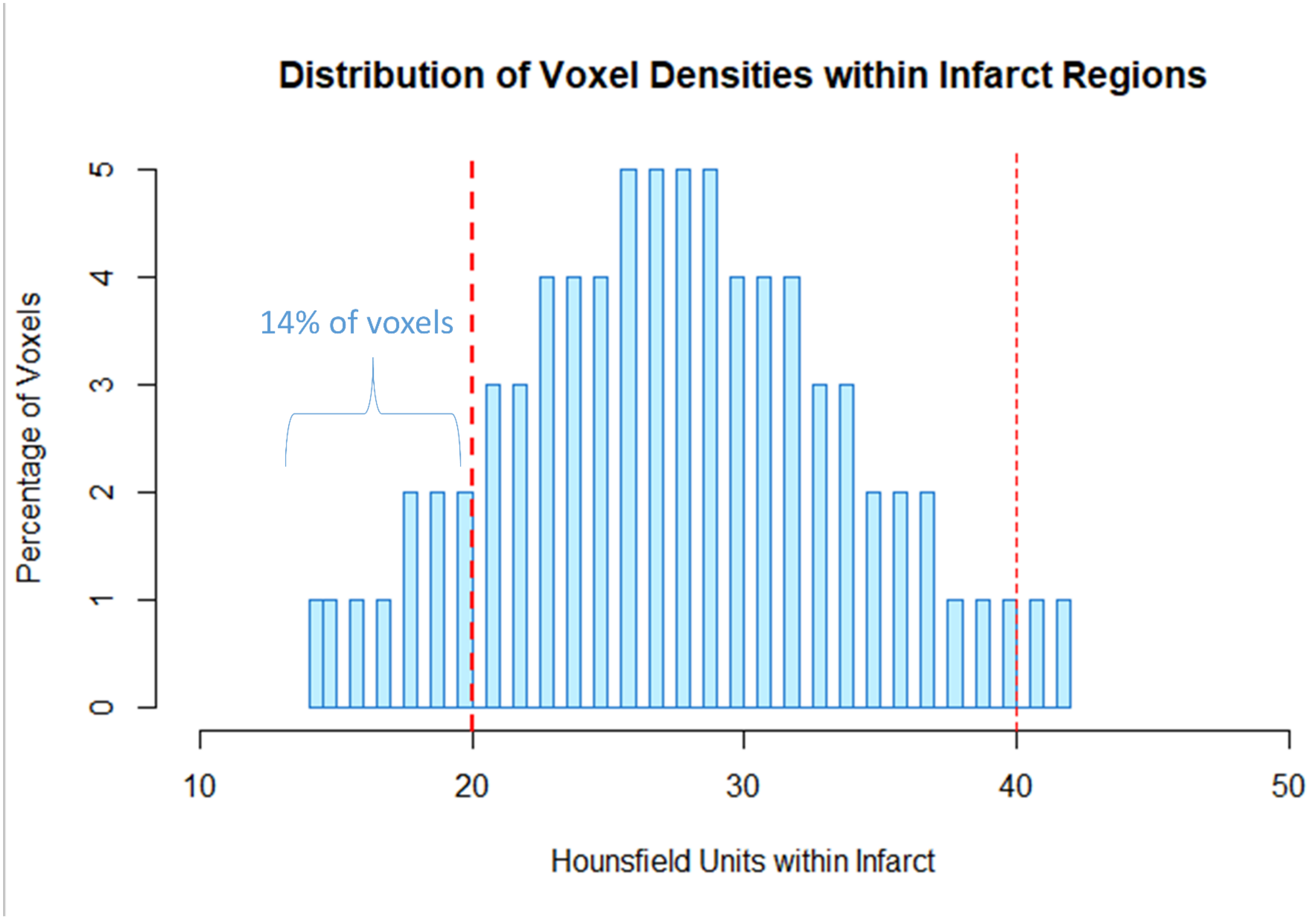

Supplemental Figure 10. Histogram of the distribution of voxel densities within infarcts in this dataset, demonstrating that 14% have density below 20 HU and that few have HU above 40.

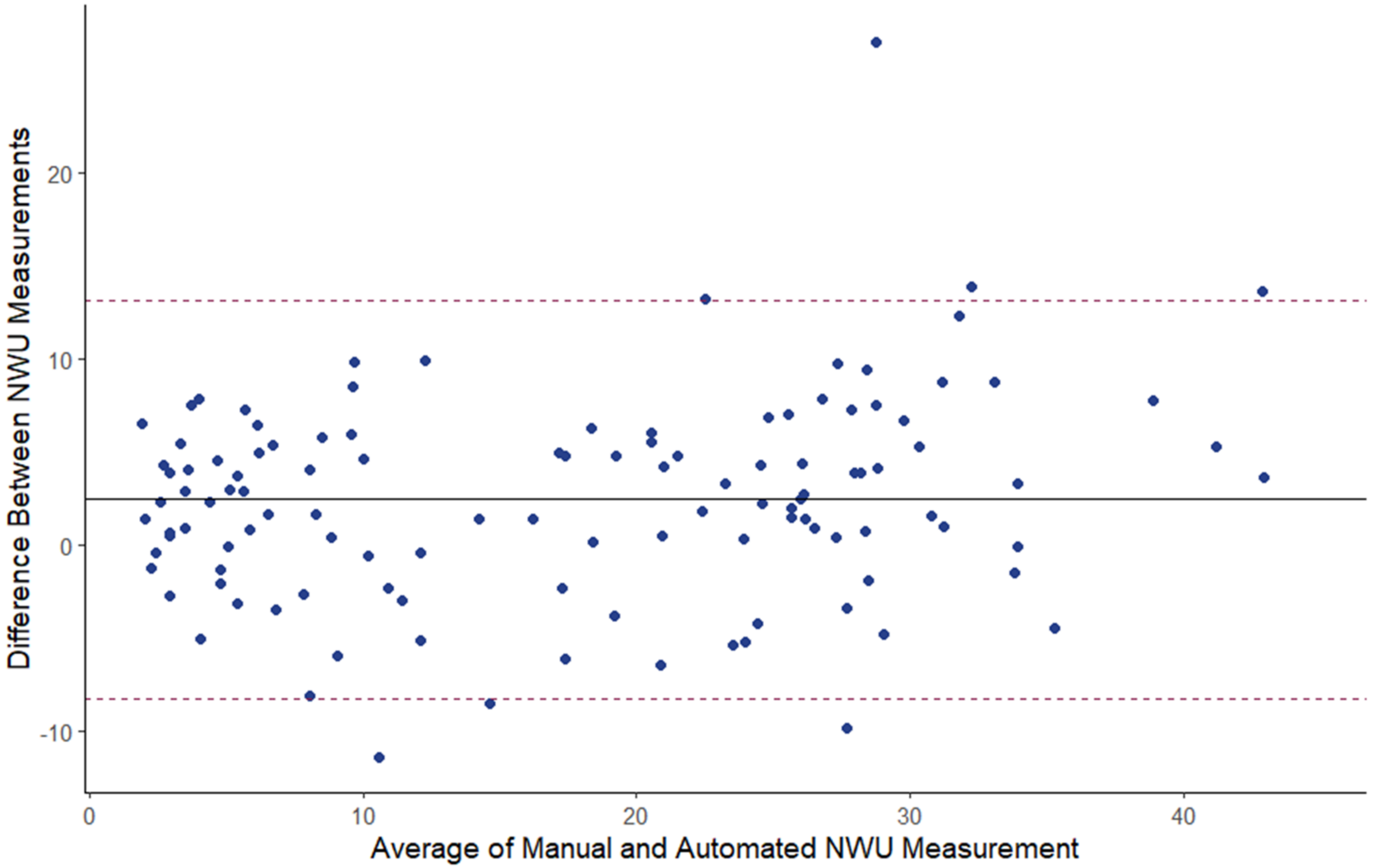

Supplemental Figure 11. Bland-Altman plot for agreement between automated and manually derived NWU values across all baseline and follow-up CTs.

REFERENCES

1. Li X, Morgan PS, Ashburner J, Smith J, Rorden C. The First Step for Neuroimaging Data Analysis: Dicom to Nifti Conversion. *J Neurosci Methods* (2016) 264:47-56. doi: 10.1016/j.jneumeth.2016.03.001.
2. Smith SM. Fast Robust Automated Brain Extraction. *Hum Brain Mapp* (2002) 17(3):143-55. Epub 2002/10/23. doi: 10.1002/hbm.10062.
3. Rorden C, Bonilha L, Fridriksson J, Bender B, Karnath HO. Age-Specific Ct and Mri Templates for Spatial Normalization. *Neuroimage* (2012) 61(4):957-65. Epub 2012/03/24. doi: 10.1016/j.neuroimage.2012.03.020.
4. Lowekamp BC, Chen DT, Ibanez L, Blezek D. The Design of Simpleitk. *Front Neuroinform* (2013) 7:45. Epub 2014/01/15. doi: 10.3389/fninf.2013.00045.
5. Jenkinson M, Bannister P, Brady M, Smith S. Improved Optimization for the Robust and Accurate Linear Registration and Motion Correction of Brain Images. *Neuroimage* (2002) 17(2):825-41. Epub 2002/10/16. doi: 10.1016/s1053-8119(02)91132-8.
6. Jenkinson M, Smith S. A Global Optimisation Method for Robust Affine Registration of Brain Images. *Med Image Anal* (2001) 5(2):143-56. Epub 2001/08/23. doi: 10.1016/s1361-8415(01)00036-6.
7. Bradski G, Kaehler A. *Learning Opencv: Computer Vision with the Opencv Library*: O'Reilly Media, Inc (2008).
8. Broocks G, Flottmann F, Scheibel A, Aigner A, Faizy TD, Hanning U, et al. Quantitative Lesion Water Uptake in Acute Stroke Computed Tomography Is a Predictor of Malignant Infarction. *Stroke* (2018) 49(8):1906-12. doi: 10.1161/STROKEAHA.118.020507.
9. Derakhshan JJ, Abedini A, Dhar R, Goyal M, McKinstry R, J.M. L. Validation of Newly Developed Ct Perfusion Software Compared to Rapid Ischemaview in 143 Patients with Acute Ischemic Stroke (Abstract). *59th Annual Meeting of the American Society for Neuroradiology*; San Francisco(2021). p. 1090.
10. Fieselmann A, Kowarschik M, Ganguly A, Hornegger J, Fahrig R. Deconvolution-Based Ct and Mr Brain Perfusion Measurement: Theoretical Model Revisited and Practical Implementation Details. *Int J Biomed Imaging* (2011) 2011:467563. Epub 2011/09/10. doi: 10.1155/2011/467563.
11. Konstas aa, Goldmakher GV, Lee T-Y, Lev MH. Theoretic Basis and Technical Implementations of Ct Perfusion in Acute Ischemic Stroke, Part 1: Theoretic Basis. *AJNR American journal of neuroradiology* (2009) 30:662-8. doi: 10.3174/ajnr.A1487.
12. Konstas AA, Goldmakher GV, Lee TY, Lev MH. Theoretic Basis and Technical Implementations of Ct Perfusion in Acute Ischemic Stroke, Part 2: Technical Implementations. *AJNR Am J Neuroradiol* (2009) 30(5):885-92. Epub 2009/03/21. doi: 10.3174/ajnr.A1492.
13. Dhar R, Chen Y, Hamzehloo A, Kumar A, Heitsch L, He J, et al. Reduction in Cerebrospinal Fluid Volume as an Early Quantitative Biomarker of Cerebral Edema after Ischemic Stroke. *Stroke* (2020) 51(2):462-7. doi: 10.1161/STROKEAHA.119.027895.
14. Ronneberger O, Fisher P, Brox T. U-Net: Convolutional Neural Networks for Biomedical Image Segmentation. In: Navab N, Hornegger J, Well W, Frangi A, editors. *Medical Image Computing and Computer-Assisted Intervention - Miccai 2015 Lecture Notes in Computer Science, Vol 9351*. Springer (2015).
15. Chen Y, Dhar R, Heitsch L, Ford A, Fernandez-Cadenas I, Carrera C, et al. Automated Quantification of Cerebral Edema Following Hemispheric Infarction: Application of a Machine-Learning Algorithm to Evaluate Csf Shifts on Serial Head Cts. *Neuroimage Clin* (2016) 12:673-80. doi: 10.1016/j.nicl.2016.09.018.
